# Adverse Health Effects from Combustion-Derived Nanoparticles: The Relative Role of Intrinsic Particle Toxicity and Host Response

**DOI:** 10.1289/ehp.0800218

**Published:** 2009-05

**Authors:** Francesco Cetta, Armand Dhamo, Laura Moltoni, Ezio Bolzacchini

**Affiliations:** Department of Surgery, Research Doctorate in Oncology and Genetics, University of Siena, Siena, Italy, E-mail: cetta@unisi.it; Department of Environmental Science, University of Milan-Bicocca, Milan, Italy

It is widely accepted that airborne pollution causes adverse health effects in humans (Gauderman et al. 2007). In addition to the concentration of particulate matter (PM), these effects have been related to innate particle toxicity. Stoeger et al. (2009) recently showed that, with a slope that significantly depends on particle structure, surface, and organic carbon content, combustion-derived nanoparticles behave in a different manner in *in vitro* systems and when reacting with lung surface (i.e. after particle–lung interaction).

We have stressed that mechanistic linkages between PM and health effects should be investigated in more detail (Cetta et al. 2007, 2008). Here, we would like to comment on the role of the individual response in the occurrence of the clinically evident outcome, that is, how individual charac teris tics of the host, in the presence of the same (or similar) noxious agents, are responsible for or determine the type and the severity of the response.

Until now, toxicity has been considered mainly as an intrinsic property of each pollutant, depending on size, type, and composition of each particle. Stoeger et al. (2009) focused specifically on structure, BET (Brunauer, Emmett, and Teller) surface area, and oxidative potency. Interestingly, they stressed particle–lung interaction and the ability of some particles rich in organic content, namely, soot with high organic content, to determine a higher than expected inflammatory response due to increased cytochrome P450 1A1 induction; they also introduced a new parameter, inflammatory efficacy, in addition to oxidative potency.

In our opinion, this could be just the top of the iceberg. In fact, “oxidative stress” is a working hypothesis in the search for a common mechanistic linkage between particulate material and adverse health effects. But it is not unique. In particular, in a recent *in vitro* study in which different types of particles were used (PM < 2.5 or = 10 μm in aero dynamic diameter, tire debris), the same concentration of different particles with the same exposure time elicited different effects on sperm cell function (motility, viability, rate of apop-tosis) (Collodel G, Geminiani M, Cetta F, Camatini M, Bolzacchini E, Renieri T, unpublished data). However, variability of the observed effects was less than that elicited by changing the host, with lower adverse effects in New Zealand rabbit sperm, more evident effects in human sperm, and very severe effects in humans with previous impaired sperm function (e.g., varicocele). Sperm cell function is easy to quantify and compare not only among different pollutants but also among different host species or subgroups. These findings are in accordance with recent epidemiologic data showing more pronounced respiratory and cardiovascular effects in patients with previous respiratory and cardiovascular impairment or specific susceptibility, respectively (Gauderman et al. 2007).

The results of these studies will also have relevant implications for policy makers (Cetta et al. 2007). In fact, until now, contrast measures have mainly been directed to reduce PM concentration. Curent evidence suggests that—more important than reducing the overall PM concentration—it is of paramount importance to reduce selectively the concentration of those pollutants that are more toxic or more strictly related with adverse health effects, such as traffic-related particles. In the future, because of the better knowledge of the host response and of the variability of individual susceptibility in the occurrence of these effects, a major goal for policy makers will be the proper and early recognition—by means of sensible and specific tests—of at-risk subpopulations.

This early recognition of at-risk subpopulations could facilitate better prevention or reduction of negative effects of host–pollutant interactions.

## Editor’s note

In accordance with journal policy, Stoeger et al. were asked whether they wanted to respond to this letter, but they chose not to do so.
